# A Meta-Regression Analysis of Utility Weights for Breast Cancer: The Power of Patients’ Experience

**DOI:** 10.3390/ijerph17249412

**Published:** 2020-12-15

**Authors:** Jiryoun Gong, Juhee Han, Donghwan Lee, Seungjin Bae

**Affiliations:** 1 College of Pharmacy, Ewha Womans University, Seoul 03760, Korea; jrkong@naver.com (J.G.); hanju1996@naver.com (J.H.); 2 Department of Statistics, Ewha Womans University, Seoul 03760, Korea; donghwan.lee@ewha.ac.kr

**Keywords:** breast cancer, utility, preferences, quality of life, meta-regression

## Abstract

To summarize utility estimates of breast cancer and to assess the relative impacts of study characteristics on predicting breast cancer utilities. We searched Medline, Embase, RISS, and KoreaMed from January 1996 to April 2019 to find literature reporting utilities for breast cancer. Thirty-five articles were identified, reporting 224 utilities. A hierarchical linear model was used to conduct a meta-regression that included disease stages, assessment methods, respondent type, age of the respondents, and scale bounds as explanatory variables. The utility for early and late-stage breast cancer, as estimated by using the time-tradeoff with the scales anchored by death to perfect health with non-patients, were 0.742 and 0.525, respectively. The severity of breast cancer, assessment method, and respondent type were significant predictors of utilities, but the age of the respondents and bounds of the scale were not. Patients who experienced the health states valued 0.142 higher than did non-patients (*p* < 0.001). Besides the disease stage, the respondent type had the highest impact on breast cancer utility.

## 1. Introduction

Breast cancer is one of the most frequent female cancers in the world, with approximately 2.09 million women newly diagnosed in 2018 [1,2]. The incidence varies across geographic locations, ranging from 18.4 per 100,000 in North Africa and the Middle East to 32.5 in Central Europe [3,4]. Breast cancer is the fifth most common cancer in Korea in 2017 [5], and the age-standardized incidence has escalated rapidly, from 26.8 per 100,000 in 1999 to 46.4 per 100,000 in 2019 [3,4].

Unlike western countries, where patients are predominantly postmenopausal [6], premenopausal patients represent approximately 15% of all patients in Korea [7], which is three times higher than that observed in western countries. Specifically, the prevalence of breast cancer is the highest in their 40s (39.7%), followed by 50s (24.98%) and 30s (14.80%) in Korea [8].

With the introduction of various surgical and non-surgical treatment options, the 5-year survival rates for non-metastatic breast cancer has been improved to as high as 92% for stage I patients [9]. Yet, these treatment options can have several negative effects on patients’ quality of life, which suggests that patients might have their life expectancy extended at the expense of their quality of life [10,11,12]. However, patients and health care providers consider both morbidity and mortality [11], so an outcome measure that incorporates both the quality and quantity of life, such as quality-adjusted life-years (QALYs), is suitable for evaluating the health outcomes of breast cancer patients [13]. Additionally, QALYs are used in the cost-utility analysis, which is a type of economic evaluation of health interventions. This is particularly relevant in cancer, where costly treatment options are frequently developed [14,15]. However, previous studies have suggested that breast cancer patients’ quality of life weights vary greatly, ranging from 0.39 to 0.61 for progressive breast cancer and from 0.52 to 0.81 for metastatic breast cancer, for example [16]. This has been frequently reported in other diseases as well [17,18,19,20,21]. These variations could be explained by the different assessment methods (direct vs. indirect methods, and subgroups of direct and indirect methods) [19,22]; types of respondents (patients allocate significantly higher utilities than does the general public) [17,21]; lower or upper bounds of scales, especially the upper anchor (the disutilities estimated from a disease-free anchor are considerably higher than those estimated from a perfect health anchor) [18,23]. Many countries have adopted economic evaluation in their reimbursement process with the introduction of various expensive drugs, where incorporating utility weights is commonly needed [24]. Although domestically obtained utility weights are generally preferred, they are not required in many jurisdictions [24], and the transferability of the utility weights are generally mixed [25]. Given that it is less feasible to obtain country-specific utility values for countries with limited infrastructure [20], providing a summary value could be very helpful for those countries.

Meta-regression has been frequently employed to summarize the QALYs to incorporate methodological heterogeneity [17,18,19]. However, it is vulnerable to false-positive results when there are too few studies or too many covariates or when methodological heterogeneity exists among the studies [26,27]. When considering breast cancer patients’ quality of life, the impacts of time since cancer diagnosis, and racial factors on utilities are inconsistent [28,29,30,31], whereas patients’ age at diagnosis and, in particular, menopausal status have been reported to consistently have a significant impact [12,32,33,34,35,36,37]. Namely, the negative impact of breast cancer on utilities was approximately four times higher for women aged 18–44 than it was for women aged ≥45 [12]. Thus, breast cancer patients’ age should be considered when estimating their preferences [12]. However, the importance of breast cancer patients’ age has been published only recently [12,36,38,39], which highlights the need to incorporate age into such analyses.

Our study has two objectives: (1) to summarize the publicly available utility values of breast cancer patients, and (2) to assess the relative impacts of study design characteristics on predicting the utilities for breast cancer. More specifically, we sought to examine whether age is a critical factor in explaining breast cancer patients’ utilities.

## 2. Materials and Methods

### 2.1. Data Collection

To identify studies reporting breast cancer patients’ utilities, we searched the Medline and Embase databases, along with Korean databases (Koreamed, RISS), for articles written in English and Korean. Korean studies were included in our analysis to better reflect the premenopausal patients’ quality of life since premenopausal patients comprise three times higher than that of western countries in Korea [7]. Studies published from January 1996 to April 2019 with the keywords “breast cancer/breast neoplasm”, “health-related quality of life”, “QALY”, “utility”, “quality-adjusted life year” and in which utilities were estimated using generic (direct and indirect) utility instruments, such as standard gamble (SG), time-tradeoff (TTO), rating scale (RS), Euroqual-5 dimension (EQ-5D), health utility instrument (HUI), short-form 6 dimension (SF-6D) and quality of well being (QWB), were included. We excluded studies that were only available as abstracts, not original research (referring to utility values obtained from previous studies, reviews, comments, or editorials), or not written in English or Korean. We also excluded utilities reporting the median rather than the mean of the utilities to be consistent with previous studies [40,41].

Utilities that had been transformed from non-preference-based, condition-specific instruments were not included in our analysis because of the methodological concerns related to mapping [42,43]. Two independent reviewers (JK, JH) reviewed the identified articles and discussed the inclusion/exclusion of the articles. Disagreement was resolved by consensus.

### 2.2. Data Extraction

We extracted the following information from the identified articles because those variables were reported to be associated with the health state utility values (HSUVs): (1) first author, name of the journal, and year of publication; (2) disease stage (early (stage 1 and 2), late (stage 3 and 4), based on the clinical definition, or not clarified) [44,45]; (3) assessment method (e.g., EQ-5D, HUI2/3, SF-6D, TTO, SG, RS); (4) respondent type (patient vs. non-patient (general public, experts, or others)) to incorporate patients’ experience with the health state utility values (HSUVs) [46,47]; (5) lower bound of the scale (death, other); (6) upper bound of the scale (perfect health, other) [23]; and (7) respondent age (non-clarified, premenopause vs. postmenopause, with 50 being the cutoff) [48,49]. If premenopausal and postmenopausal respondents are mixed together, then it was classified as “not clarified”.

Breast cancer surgery (mastectomy or other) was not considered because it was not significant in a previous meta-regression analysis [40], and more than half of the utilities did not clarify surgery status (data not shown). Other variables (chemotherapy, response to treatment) were not included in our analyses because clear information was not available in many studies.

### 2.3. Statistical Analysis

To incorporate heterogeneity into the study design, we conducted a meta-regression using a hierarchical linear model (HLM), which is a mixed-effects regression model for nested designs [50]. A single study is likely to contain more than one utility estimate; thus, those values are likely to be correlated. This violates the assumption of independence necessary for a traditional ordinary least square regression model. In our HLM model, each utility value is treated as being nested within a cluster, which is the study the utility is being reported.

We included six explanatory variables in the model: disease stage, assessment method, types of the respondent, administration method, lower and upper bounds of the scale, and the age of the respondent, with the utility being the dependent variable. The statistical significance of the predictor variables was assumed if the *p*-value was lower than 0.05. The model employed a random intercept for each study, with a common slope used across all groups [18,51].

The reference case for the model was early-stage breast cancer, postmenopausal, rated by non-patients (own health) using TTO, interviewed with a scale ranging from death to perfect health.

The utility values were not transformed because they showed a normal distribution, which is consistent with a previous study [18]. Thus, data transformation was not required. Administration method was frequently missing in many studies; we included those studies with unspecified administration methods in the analysis to yield as many samples as possible. Statistical analysis was performed using the Proc Mixed procedures in SAS version 9.4 (SAS Institute Inc., Cary, NC, USA).

## 3. Results

### 3.1. Literature Review

**Figure 1:**
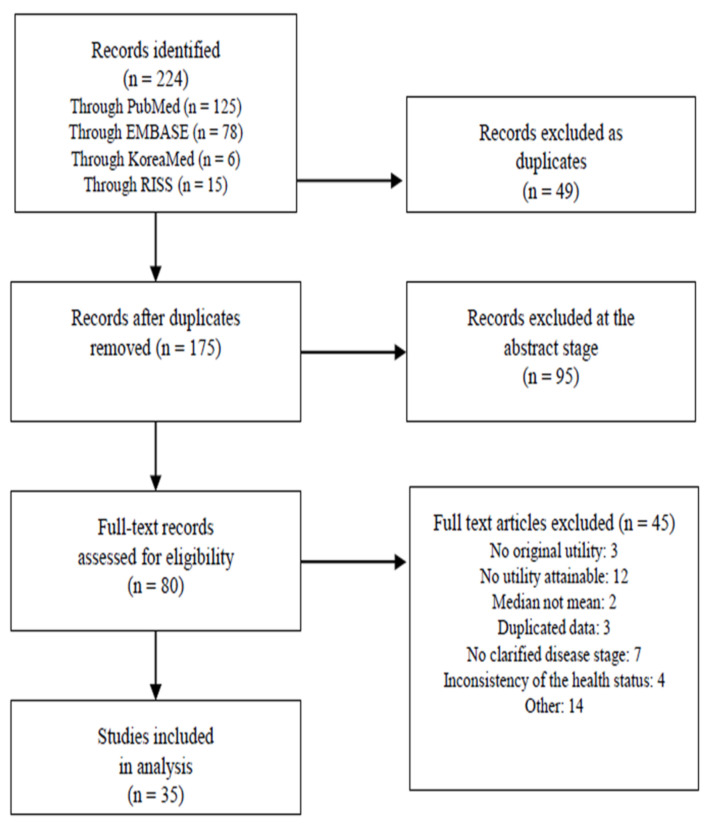
Flow diagram of the systematic review of breast cancer utility studies.

We initially identified 224 studies reporting QALYs for breast cancer, and after excluding 49 duplicate studies, we reviewed 175 abstracts. Ninety-five studies were excluded based on the abstracts, leaving 80 studies for full-text review. An additional 45 of those studies were excluded because they did not clarify the disease state [32,52,53,54,55,56,57,58] or were redundant with previous [59,60,61,62] studies where utilities were not clearly reported [63,64,65,66,67,68,69,70,71,72,73,74,75,76,77], leaving 35 studies [16,31,37,78,79,80,81,82,83,84,85,86,87,88,89,90,91,92,93,94,95,96,97,98,99,100,101,102,103,104,105,106,107,108,109] for our analysis (Table 1, Figure 1).

### 3.2. Study Characteristics

**Figure A1:**
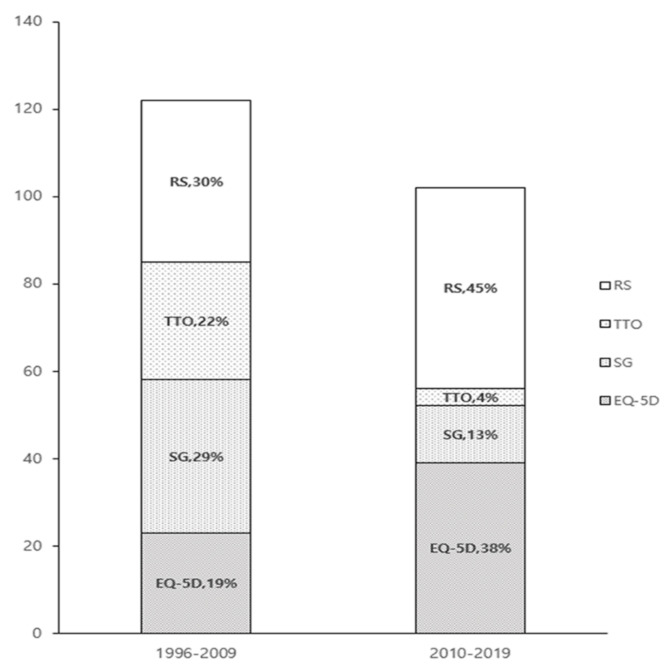
Percentage of assessment methods used in the studies from January 1996 to April 2019 (total *n* = 224). Year 1996~2009, *n* = 122; year 2010~2019, *n* = 102; SG = standard gamble; RS = rating scale; TTO = time-tradeoff; EQ-5D = Euroqual-5 dimension

Of those 35 studies, 18 had been published since 2010 [16,31,78,79,80,82,85,86,88,91,94,95,96,103,104,106,108,109] (Table 1). 16 of the 35 studies were conducted in Europe, and 10 in Asia. Frequently, the age of the participants was not clearly divided based on the menopausal status (Table 1). Overall, 224 utility values were collected in the 35 studies (Table 2). Of those 224 utilities, 38.8% evaluated early-stage (*n* = 87), while 31.3% (*n* = 70) corresponded to stages III and IV, and 29.9% of them did not specify the disease states (Table 2). RS was most frequently used (37.1%, *n* = 83), followed by EQ-5D (27.7%, *n* = 62) and SG (21.4%, *n* = 48). EQ-5D and RS were more likely to be used in studies published after 2010 (38% and 45% of the utilities, respectively) than in studies published before 2010 (19% and 30% of the utilities, respectively). However, the use of SG and TTO plummeted from 29% and 22% in the earlier period to 13% and 4% in the later period, respectively (Figure A1).

Patients served as respondents in approximately 68.3% (*n* = 153) of the utilities, and younger (age < 50) respondents represented 16.1% of the respondent (*n* = 36, Table 2). 20.1% of the utilities defined the lower and upper anchors as death to perfect health, with “other” included best/worst possible health, good/full health, disease-free state, or anchors were not stated/defined. The quality of the included study was evaluated by Papaioannou and colleagues’ study [110]; loss to follow-up information was frequently missing, and the sample size and response rates varied widely. (Appendix B).

### 3.3. Regression Analysis

**Figure 2:**
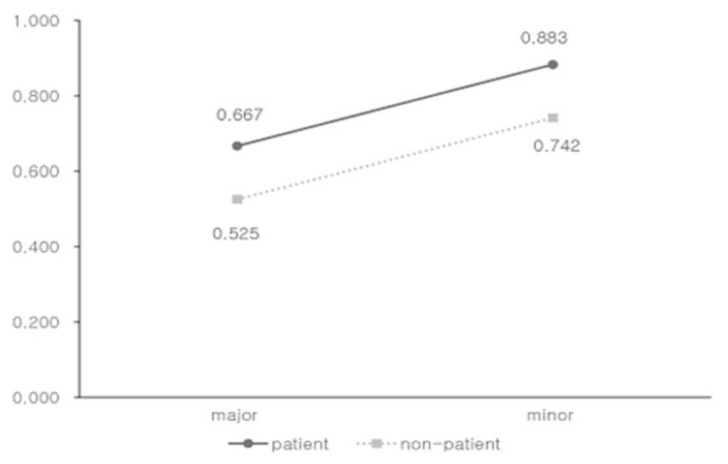
Estimated utility values for breast cancer stages stratified by respondent type, using the time-tradeoff with the scales anchored by death to perfect health.

The results of the HLM model are shown in Table 3. We estimated the utility for the reference case for the early and late-stage (using the TTO with the scales anchored by death to perfect health estimated by non-patients) to be 0.742 and 0.525, respectively. Our analysis showed that the disease stage, assessment method, and types of respondents were significant predictors of utilities (*p* < 0.05). However, the lower and upper anchors of the scales and respondent age were not significant predictors. Specifically, the elicitation methods were significantly associated with the HSUVs, with TTO being highest (0.135 higher than those estimated from RS, holding constant all other variables), followed by SG and EQ-5D, and with RS being the lowest. Also, the response type was an important predictor; patients who experienced the health states valued 0.142 higher than did non-patients (*p* < 0.001, Figure 2). Age was not significantly associated with the HSUVs.

We conducted a subgroup analysis including 10 Asian studies [78,79,80,82,85,94,95,105,106,109], and the results were consistent with the main results in terms of the impact and the statistical significance of the respondent type (Table 4).

## 4. Discussion

In this meta-regression analysis, we pooled 224 HSVs from 35 studies and estimated the breast cancer utility values of 0.742 and 0.525 for early- and late-stage breast cancer, respectively, using non-patients as the respondents, TTO as the elicitation method, lower and upper bounds of the scale as the death to perfect health and others, and the respondent age being 50 years and older.

We found that the disease stage was significantly associated with breast cancer utility values, which is consistent with previous studies [17,18,19]. Interestingly, the utilities estimated from TTO were the highest, followed by SG, EQ-5D, and RS, in the main model, while holding other variables constant. However, it has been reported that the values estimated from SG were usually highest, followed by TTO and RS [22,111]. The order of TTO and SG was switched in our study, as observed in other studies [19,40]. The difference between TTO and SG, however, was marginal (0.037), consistent with previous studies [37]. Our results also showed that the TTO values were higher than the EQ-5D values, as seen in previous studies [40,89]. Lidgren and colleagues reported that early-stage breast cancer patients were not willing to trade their life expectancy for improved quality of life, even though their current health status was far from perfect [89]. The authors speculated that those patients might assume their full recovery after treatment, which would improve their quantity and quality of life. Late-stage breast cancer patients might not be willing to trade their life expectancy at all; this is known as the violation of the constant proportional assumption and is prevalent in patients with limited life expectancy [112]. Thus, it is not surprising that the values obtained from TTO were higher than those obtained from EQ-5D or SG in breast cancer patients of all stages.

Our results illustrated that EQ-5D has been more frequently employed in studies published since 2010 (Appendix A). This might be associated with the fact that the UK NICE recommended the use of the EQ-5D as a reference case in 2008 [113,114], and Australian PBAC has stipulated that indirect preference-based, multi-attribute utility instruments (MAUIs) such as HUI, EQ-5D, and SF-6D are preferred [115]. In Korea, EQ-5D is the only MAUI that has a tariff for the general Korean population, and the Korean guidelines specified that preference should be preferably sourced from the domestic, general population, which explains the increased use of EQ-5D [116]. If appropriate elicitation methods for breast cancer were recommended based on the practicality, validity, and reliability [117], then the comparability and homogeneity among breast cancer studies would be enhanced.

Our study also suggested that the type of respondent had the highest impact on the utility, holding other variables constant. The utilities derived from patients were higher than non-patients in every condition, which is consistent with previous studies [22]. It is not surprising that the patient’s experience is the most significant predictor in the breast cancer utility estimate, since patients’ experience may influence more for those diseases with long life expectancy and good treatment options, such as breast cancer. Our finding is consistent with what has been discussed in the assessment methods, with breast cancer patients not willing to trade their life expectancy regardless of the disease stage. Although future study is needed, our analysis suggested that patients’ experience may count more for breast cancer patients, thus, special attention should be paid to selecting respondents.

Additionally, although the age of the respondent was reported to be significantly associated with the HSUVs by Brown and colleagues [12], with younger patients reporting lower HSUVs compared with their older counterparts, which is consistent with our study, statistical significance was still not achieved, which might be related with the fact that the age of the respondent was frequently not clarified (Table 2). Further study is required.

Our study sought to summarize breast cancer utility values by including relevant variables (age) and being limited to studies with clear information that could improve the reliability of the results considerably [27]. However, our study suffered the following limitations. First, insufficient information was available regarding the definition of the health states; we tried to keep as many studies as possible while having clear definitions of the health states, which forced us to classify the health states rather crudely (i.e., early-stage, late-stage). Information about the treatment type (types of surgery, types of chemotherapy) was also frequently missing in the original data, so although the treatment type could influence the HSVs, we could not include that variable in our analysis. In addition, the sample size and response rate of the included studies varied greatly, and little information was available regarding missing data, loss-to-follow up, or evaluating the appropriateness of the measure. Also, there was only one study using HUI3, which reported two health state values [97], which was excluded due to statistical concerns. We conducted sensitivity analyses including the study which used HUI3, and we categorized HUI3 and EQ-5D together as “MAUI”, and the results were still consistent with those of our main model. Finally, our base case analysis was based on the mean rather than the median of the utilities to be consistent with previous studies [41]. We conducted sensitivity analyses with studies reporting medians instead of means, and the results were consistent with our analysis.

## 5. Conclusions

The present study summarized the quality of life weights for breast cancer patients and demonstrated that disease severity, elicitation method, and response type were significantly associated with the weights but that the respondent age and scale bounds were not. Our analysis suggested that the respondent had the highest impact on the quality of life weights, and special attention should be paid to patients’ experience when estimating utility for breast cancer.

## Figures and Tables

**Table 1:** Study characteristics.

Reference	Location	Disease Stage	Assessment Method	Respondent Type	Lower and Upper Bound of the Scale	Age
Grann (1998)	USA	Early-stage/ Late-stage	TTO	Non-patient	Other	Premenopausal
Hürny (1998)	Switzerland	Late-stage	TTO	Patient	Death to Perfect health	Not-stated
Jansen (1998)	Netherlands	Early-stage	SG/TTO	Patient	Other	Not-stated
Grann (1999)	USA	Not-stated	TTO/RS	Patient/ Non-patient	Other	Not-stated/ Premenopausal
Chie (2000)	Taiwan	Early-stage/ Late-stage	RS	Non-patient	Death to Perfect health	Not-stated
Jansen (2000)	Netherlands	Early-stage	SG/TTO/RS	Patient	Death to Perfect health or Other	Premenopausal
Jansen (2000)	Netherlands	Early-stage	SG/TTO/RS	Patient	Death to Perfect health or Other	Not-stated
Polsky (2002)	USA	Early-stage	RS	Patient	Other	Postmenopausal
Jansen (2004)	Netherlands	Early-stage	EQ-5D/RS	Patient	Death to Perfect health or Other	Not-stated
Conner-Spady (2005)	Canada	Late-stage	EQ-5D/RS	Patient	Death to Perfect health or Other	Not-stated
Lloyd (2006)	UK	Late-stage	SG	Non-patient	Other	Not-stated
Milne (2006)	New Zealand	Late-stage	EQ-5D/TTO/RS	Non-patient	Other	Not-stated
Schleinitz (2006)	USA	Early-stage/ Late-stage	SG	Non-patient	Death to Perfect health	Not-stated
Lidgren (2007)	Sweden	Early-stage/ Late-stage	EQ-5D/TTO	Patient	Other	Not-stated
Mansel (2007)	UK	Early-stage	SG	Non-patient	Death to Perfect health	Postmenopausal
Buyukdamgaci-Alogan (2008)	Tukey	Early-stage	SG	Non-patient	Other	Premenopausal
Kimman (2009)	Netherlands	Not-stated	EQ-5D/RS	Patient	Other	Not-stated
Freedman (2010)	USA	Early-stage	EQ-5D/RS	Patient	Other	Not-stated/Premenopausal/ Postmenopausal
Haines (2010)	Australia	Not-stated	EQ-5D/RS	Patient	Other	Not-stated
Kimman (2011)	Netherland	Not-stated	EQ-5D	Patient	Other	Not-stated
Cheng (2012)	Taiwan	Early-stage	SG	Patient	Other	Not-stated
Kim (2012)	Korea	Late-stage	EQ-5D	Patient	Death to Perfect health	Not-stated
Shih (2012)	Singapore	Not-stated	RS	Non-patient	Other	Not-stated
Frederix (2013)	Netherland	Early-stage/ Late-stage	TTO/RS	Patient	Death to Perfect health	Postmenopausal
Moro-Valdezate (2013)	Spain	Not-stated	EQ-5D	Patient	Other	Not-stated
Farkkila (2014)	Finland	Late-stage	EQ-5D/RS	Patient	Other	Not-stated
Matter-Walstra (2014)	Switzerland	Not-stated	EQ-5D/RS	Patient	Other	Not-stated/Premenopausal/ Postmenopausal
Tan (2014)	Singapore	Early-stage/ Late-stage	SG/RS	Patient	Death to Perfect health or Other	Not-stated
Kim (2015)	Korea	Not-stated/ Early-stage/ Late-stage	EQ-5D/RS	Patient	Other	Not-stated/Premenopausal/ Postmenopausal
Kim (2015)	Korea	Not-stated	RS	Patient	Other	Not-stated
Luo (2015)	Singapore	Not-stated	EQ-5D	Patient	Other	Not-stated
Pickard (2016)	USA	Late-stage	EQ-5D/RS	Patient	Other	Not-stated
Kim (2017)	Korea	Early-stage/ Late-stage	SG/RS	Non-Patient	Other	Not-stated
Rautalin (2018)	Finland	Late-stage	EQ-5D/RS	Patient	Other	Not-stated
Li (2019)	China	Not-stated	EQ-5D/TTO	Patient	Other	Premenopausal/ Postmenopausal

SG = standard gamble; RS = rating scale; TTO = time-tradeoff; EQ-5D = Euroqual-5 dimension. Other includes best/worst possible health, good/full health, disease-free state, or the definitions of the anchors were not available. The country of the study was classified based on the participants’ information if available, and authors’ affiliations if participants’ information is not available. If authors have multiple affiliations, the corresponding authors’ country was selected.

**Table 2:** Characteristics of the utility weights by disease stage, assessment method, respondent type, survey origin, scale bounds, and administration method.

Variable		Number of Utilities (%)	Number of Studies	References
Disease stage	Not-stated	67 (29.9)	11	[78,80,85,86,88,94,96,99,100,104,106]
	Late-stage	70 (31.3)	16	[16,37,79,83,89,90,91,93,95,101,103,105,106,107,108,109]
	Early-stage	87 (38.8)	17	[16,31,37,79,81,82,83,84,87,89,92,97,98,102,105,106,109]
Assessment method	EQ-5D	62 (27.7)	17	[31,80,86,88,89,91,92,93,94,95,96,100,103,104,106,107,108]
	SG	48 (21.4)	10	[37,79,81,82,84,87,90,98,102,109]
	TTO	31 (13.8)	10	[16,80,83,87,89,98,99,101,102,107]
	RS	83 (37.1)	21	[16,31,78,79,85,87,91,92,93,96,97,99,100,102,103,104,105,106,107,108,109]
Respondent type	Patient	153 (68.3)	26	[16,31,78,79,80,82,86,87,88,89,91,92,93,94,95,96,97,98,99,100,101,102,103,104,106,108]
	Non-patient	71 (31.7)	10	[37,81,83,84,85,90,99,105,107,109]
Survey origin	Not-stated	3 (1.3)	1	[83]
	Scenario	125 (55.8)	14	[16,37,79,81,84,85,90,98,99,100,102,105,107,109]
	Own health	96 (42.9)	20	[31,62,78,80,82,86,87,88,89,92,93,94,95,96,97,101,103,104,106,108]
Administration method	Not-stated	21 (9.4)	4	[81,83,84,94]
	Self	92 (41.1)	15	[31,78,88,89,91,92,93,94,95,99,103,104,105,106,108]
	Interview	111 (49.5)	18	[16,37,79,80,82,85,86,87,90,94,96,97,98,100,101,102,107,109]
Low and upper bounds of the scales	Others	179 (79.9)	29	[31,78,79,80,82,83,84,85,86,87,88,89,90,91,92,93,94,96,97,98,99,100,102,103,104,106,107,108,109]
	Perfect health/ Death	45 (20.1)	11	[16,37,79,81,87,92,93,95,101,102,105]
Age	Not-stated	166 (74.1)	28	[31,37,78,79,82,85,86,88,89,90,91,92,93,94,95,96,98,99,100,101,102,103,104,105,106,107,108,109]
	<50	36 (16.1)	8	[31,80,83,84,87,99,104,106]
	≥50	22 (9.8)	7	[16,31,80,81,97,104,106]

SG = standard gamble; RS = rating scale; TTO = time-tradeoff; EQ-5D = Euroqual-5 dimension. Other includes best/worst possible health, good/full health, disease-free state, or the definitions of the anchors were not available.

**Table 3:** The result of the HLM model (*n* = 224): coefficient estimates, *p*-values, and 95% confidence intervals (CI) for the predictors of utilities.

Variables	Coefficient Estimates	95% CI	*p*-Value
Intercept	0.742	0.624, 0.859	<0.001
**Disease stage**			
Not-stated	−0.039	−0.115, 0.038	0.321
Late-stage	−0.216	−0.282, −0.150	<0.001
Early-stage	Reference	Reference	Reference
**Assessment method**			
EQ-5D	−0.065	−0.140, 0.009	0.084
SG	−0.037	−0.113, 0.038	0.329
RS	−0.135	−0.203, −0.068	<0.001
TTO	Reference	Reference	Reference
**Respondent type**			
Patient	0.142	0.065, 0.218	<0.001
Non-Patient	Reference	Reference	Reference
**Lower and upper bounds of the scales**			
Others	0.014	−0.051, 0.078	0.676
Death to perfect health	Reference	Reference	Reference
**Age**			
Not-stated	0.003	−0.077, 0.083	0.936
<50 (Premenopausal)	−0.006	−0.098, 0.086	0.897
≥50 (Postmenopausal)	Reference	Reference	Reference

SG = standard gamble; RS = rating scale; TTO = time-tradeoff; EQ-5D = Euroqual-5 dimension. Other includes best/worst possible health, good/full health, disease-free state, or the definitions of the anchors were not available.

**Table 4:** The result of the HLM model (*n* = 75, only including Asian countries): coefficient estimates, *p*-values, and 95% confidence intervals (CI) for the predictors of utilities.

Variables	Coefficient Estimates	95% CI	*p*-Value
Intercept	0.741	0.436, 0.890	<0.001
**Disease stage**			
Not-stated	−0.056	−0.146, 0.032	0.209
Late-stage	−0.264	−0.342, −0.186	<0.001
Early-stage	Reference	Reference	Reference
**Assessment method**			
EQ-5D	0.033	−0.153, 0.219	0.728
SG	−0.012	−0.224, 0.202	0.916
RS	−0.114	−0.309, 0.082	0.249
TTO	Reference	Reference	Reference
**Respondent type**			
Patient	0.165	0.019, 0.3311	0.027
Non-Patient	Reference	Reference	Reference
**Lower and upper bounds of the scales**			
Others	−0.052	−0.179, 0.075	0.413
Death to perfect health	Reference	Reference	Reference
**Age**			
Not-stated	−0.004	−0.111, 0.105	0.923
<50 (Premenopausal)	0.005	−0.111, 0.121	0.931
≥50 (Postmenopausal)	Reference	Reference	Reference

SG = standard gamble; RS = rating scale; TTO = time-tradeoff; EQ-5D = Euroqual-5 dimension. Other includes best/worst possible health, good/full health, disease-free state, or the definitions of the anchors were not available.

## Appendix B. Quality Evaluation of the Included Studies

**Reference**	**Sample Size**	**Respondent** **Selection and Recruitment**	**Inclusion/** **Exclusion Criteria**	**Response Rates to the Instrument Used**	**Loss to Follow-Up**	**Missing Data**	**Any Other Problems with the Study**
Grann (1998)	54	not reported	Yes	not repoted	No follow-up	not repoted	small samples for generalization
Hürny (1998)	83	Yes	Yes	100%	No follow-up	not reported	population (patient) from various countries and cultures
Jansen (1998)	70	Yes	Yes	36%	No follow-up	7%	small samples for generalization
Grann (1999)	135	Yes	Yes	not reported	No follow-up	not reported	representative of the health states
Chie (2000)	979	Yes	Yes	not reported	No follow-up	not reported	low response rate
Jansen (2000)	41	Yes	Yes	76%	24%	not reported	small sample for generalization
Jansen (2000)	70	Yes	Yes	64%	22%	not reported	small sample for generalization
Polsky (2002)	784	Yes	Yes	68%	not reported	5%	selection bias for generalization
Jansen (2004)	448	Yes	Yes	62%	No follow-up	10%	selection bias for generalization
Conner-Spady (2005)	52	Yes	Yes	92%	14%	not reported	small sample for generalization
Lloyd (2006)	106	Yes	not reported	not reported	No follow-up	6%	health state validation unclear
Milne (2006)	50	Yes	Yes	not reported	No follow-up	No	small sample for generalization
Schleinitz (2006)	156	Yes	Yes	78%	No follow-up	not reported	language difference
Lidgren (2007)	345	Yes	Yes	96%	No follow-up	6%	selection bias
Mansel (2007)	not reported	not reported	Yes	not reported	not reported	not reported	lack of information
Buyukdamgaci-Alogan (2008)	30	Yes	Yes	not reported	No follow-up	not reported	small sample for generalization
Kimman (2009)	192	Yes	Yes	87%	no follow-up	not reported	small subgroup analysis for generalization
Freedman (2010)	1050	Yes	Yes	not reported	54%	not reported	representative of the health states
Haines (2010)	89	Yes	Yes	76%	18%	not reported	low response and follow-up rate
Kimman (2011)	299	Yes	Yes	not reported	12%	1%	selection bias
Cheng (2012)	152	Yes	Yes	not reported	No follow-up	not reported	large amount of missing data
Kim (2012)	199	Yes	Yes	100%	no follow-up	no exist	selection bias for generalization
Shih (2012)	20	Yes	Yes	61%	No follow-up	no exist	small sample for generalization
Frederix (2013)	200	Yes	Yes	not reported	No follow-up	No exist	representative of the health states
Moro-Valdezate (2013)	364	Yes	Yes	66%	34%	Not reported	lack of baseline HRQOL measurements prior to treatment
Farkkila (2014)	27	Yes	Yes	not reported	No follow-up	not reported	small sample for generalization
Matter-Walstra (2014)	92	Yes	Yes	not reported	No follow-up	No exist	language difference
Tan (2014)	64	Yes	Yes	68%	No follow-up	not reported	small sample
Kim (2015)	827	Yes	Yes	83%	No follow-up	21%	generalizability (one hospitals selected)
Kim (2015)	299	Yes	Yes	90%	No follow-up	not reported	representative of the health states
Luo (2015)	269	Yes	Yes	94%	6%	1%	Selection bias
Pickard (2016)	50	Yes	Yes	not reported	No follow-up	No exist	small sample
Kim (2017)	509	Yes	Yes	not reported	No follow-up	not reported	selection bias
Rautalin (2018)	840	Yes	Yes	59%	No follow-up	No exist	low response rate
Li (2019)	608	Yes	Yes	not reported	No follow-up	2%	selection bias
